# Modeling seizure networks in neuron-glia cultures using microelectrode arrays

**DOI:** 10.3389/fnetp.2024.1441345

**Published:** 2024-09-03

**Authors:** Ujwal Boddeti, Jenna Langbein, Darrian McAfee, Marcelle Altshuler, Muzna Bachani, Hitten P. Zaveri, Dennis Spencer, Kareem A. Zaghloul, Alexander Ksendzovsky

**Affiliations:** ^1^ Surgical Neurology Branch, NINDS, National Institutes of Health, Baltimore, MD, United States; ^2^ Department of Neurosurgery, University of Maryland School of Medicine, Baltimore, MD, United States; ^3^ Department of Neurosurgery, Brigham and Women’s Hospital, Boston, MA, United States; ^4^ Department of Neurology, Yale University, New Haven, CT, United States; ^5^ Department of Neurosurgery, Yale University, New Haven, CT, United States

**Keywords:** 4-aminopyridine, epilepsy, epilepsy model, functional connectivity, microelectrode arrays, network changes, neuronal networks, seizure networks

## Abstract

Epilepsy is a common neurological disorder, affecting over 65 million people worldwide. Unfortunately, despite resective surgery, over 30 % of patients with drug-resistant epilepsy continue to experience seizures. Retrospective studies considering connectivity using intracranial electrocorticography (ECoG) obtained during neuromonitoring have shown that treatment failure is likely driven by failure to consider critical components of the seizure network, an idea first formally introduced in 2002. However, current studies only capture snapshots in time, precluding the ability to consider seizure network development. Over the past few years, multiwell microelectrode arrays have been increasingly used to study neuronal networks *in vitro*. As such, we sought to develop a novel *in vitro* MEA seizure model to allow for study of seizure networks. Specifically, we used 4-aminopyridine (4-AP) to capture hyperexcitable activity, and then show increased network changes after 2 days of chronic treatment. We characterize network changes using functional connectivity measures and a novel technique using dimensionality reduction. We find that 4-AP successfully captures persistently elevated mean firing rate and significant changes in underlying connectivity patterns. We believe this affords a robust *in vitro* seizure model from which longitudinal network changes can be studied, laying groundwork for future studies exploring seizure network development.

## Introduction

Epilepsy affects over 65 million people worldwide (Ngugi et al., 2010). When patients fail medical management with anti-seizure medications (ASMs), they must turn to surgical evaluation. However, despite resective surgery and/or neuromodulatory intervention, approximately 50 % of patients experience refractory seizures (Andrews et al., 2019). Recent studies considering electrocorticography (ECoG) obtained during neuromonitoring have suggested that treatment failure is likely attributed to untreated components of a pathologic seizure network (Boddeti et al., 2022). Although there are numerous preexisting *in vitro* and *in vivo* seizure models, to our knowledge, none model seizure network development. As such, in this study, we describe a novel *in vitro* epilepsy model using multiwell microelectrode arrays (MEAs), to study seizure network development.

Epilepsy models suggest that seizures are characterized by excessive synchronous neuronal firing. Primary epilepsies (i.e., genetic epilepsies) are modeled by knock-out mice (ex. Kcnq3, Lgi1, Mecp2, etc) and secondary epilepsies are modeled using induction protocols (Marshall et al., 2021). These typically involve kindling via electrical stimulation or chemoconvulsant agents (ex. Pilocarpine, Pentylenetetrazole (PTZ), Kainate) that are introduced on repeated occasions, resulting in eventual spontaneous seizures (Curia et al., 2008; Lévesque and Avoli, 2013; Dhir, 2012; Goddard, 1967). Electrophysiology and seizure activity are typically recorded using invasive cortical/subcortical electrodes (Marshall et al., 2021).

In contrast to animal models, *in vitro* epilepsy models typically consist of mammalian brain slices, derived from whole brain or hippocampus, for acute or chronic (organotypic slices) study (Raimondo et al., 2017). These slices are obtained either from animal models of epilepsy or naive-animals. Electrophysiology from slices is recorded using a number of options, including grease gas chambers, ion-selective microelectrodes, functional microscopy and optogenetics, and most commonly, patch-clamp (Raimondo et al., 2017; Neher and Sakmann, 1976; Sakmann and Neher, 1984). Patch-clamp is an electrophysiological technique developed in the 1970s that allows for study of single-neuron electrical behavior using a micropipette in tight contact with the cell membrane (Segev et al., 2016). Patch-clamp recordings afford many advantages, including the ability to record from neurons in the context of their native, preserved circuitry and also capturing changes in activity in real-time. However, by virtue of how patch-clamp recordings are obtained, activity across multi-unit neuronal populations over long periods of time are not feasible. Furthermore, the quality of recordings from patch-clamp can vary greatly depending on cell-specific conditions, presenting a further challenge (Saleem et al., 2023). As such, *in vitro* slice models and patch-clamp recordings do not allow for effective study of network activity or changes.

Over the past few years, MEA technology has been increasingly used for *in vitro* investigations to better understand neuronal dynamics, balancing neuron-glia populations and a well-controlled environment to model neurologic disease (Mossink et al., 2021; Cerina et al., 2023). MEAs encompass microelectrodes at the base of tissue culture wells, on which neuronal populations are plated. These microelectrodes allow for capture of high-frequency neuronal spiking activity by recording of extracellular field potential from multiple electrodes, which studies have shown strongly resembles intracellular waveforms (Ashida et al., 2012; Funabiki et al., 2011; Kuokkanen et al., 2018). Additionally, MEA microelectrodes allow for delivery of electrical stimulation, allowing for intentional probing of *in vitro* neuronal networks. As discussed above, traditional *in vitro* epilepsy models use patch-clamp recordings from brain slices. Although patch-clamp directly captures intracellular neuronal action potentials, it fails to capture activity from populations of neurons. MEAs offer a unique advantage as recordings from extracellular field potential allow for useful estimates of population activity. In recent years, MEAs have been used to model neurological disease, such as Alzheimer’s disease (AD), Glioblastoma Multiforme (GBM), Parkinson’s disease (PD), Amyotrophic Lateral Sclerosis (ALS), and epilepsy (Li et al., 2020; Krishna et al., 2023; Wainger et al., 2014; Woodard et al., 2014; Tidball and Parent, 2016). Considering increasing evidence suggesting pathomechanisms surrounding epileptogenesis reflect distributed seizure networks, MEAs offer a robust tool in which such changes can be studied and modeled.

In a cohort of patients with drug-resistant epilepsy (DRE), we found evidence of increased functional connectivity (FC) between regions of seizure onset and spread. Based on these findings, it is plausible that years of repeated seizure insults may result in changes in underlying neuronal connectivity, that result in interictal FC changes. As such, to better understand network changes that may take place in epilepsy patients, we describe an *in vitro* epilepsy model, motivated by our findings in DRE patients, where we capture seizure-like hyperexcitability over time and monitor network changes longitudinally. We use 4-aminopyridine (4-AP), a known pro-convulsant agent, to capture hyperexcitable activity and model seizures. We use electrophysiology recorded from MEAs to characterize network changes after 2 days of chronic 4-AP treatment. We posited that we would be able to effectively capture network changes in our seizure model, serving as a proof-of-concept that our *in vitro* MEA seizure model could be used to study seizure network development over time. We find that 4-AP successfully captures hyperexcitable activity that persists at baseline. Furthermore, we find after 2 days of chronic treatment, network connectivity is significantly increased in 4-AP treated wells, compared to control, when considering functional connectivity (FC) and a novel technique using dimensionality reduction. These results support the idea that 4-AP can be used to effectively model hyperexcitable activity on MEAs and allow for *in vitro* study of network changes, that may provide insight into the pathogenesis of seizure networks.

## Materials and methods

### Functional connectivity in human epilepsy patients

We retrospectively identified three patients (1 female, 39.0 ± 13.1 years, ( $\bar{x}$ ± σ ), with mesial-temporal lobe epilepsy (MTLE) who underwent resective surgery. All patients in the study cohort underwent neuromonitoring between 1 August 2014, and 1 November 2017, for 11.7 ± 4.2 days ( $\bar{x}\;\pm \;\sigma$ ). In each case, the clinical team determined placement of electrode contacts to localize ictal regions. All surgical procedures and icEEG monitoring were performed at the Yale Comprehensive Epilepsy Center at the Yale New Haven Hospital (YNHH) (New Haven, CT). During neuromonitoring, each patient had 4.0 ± 2.0 seizures ( $\bar{x}\;\pm \;\sigma$ ), for a total of 12 seizures across all patients, and 4.0 ± 1.0 ( $\bar{x}\;\pm \;\sigma$ ) interictal epochs were extracted. The study was conducted with approval from the Yale Institutional Review Board (IRB) and informed consent was obtained from all participants. The study was conducted in accordance with the relevant guidelines and regulations.

**FIGURE 1:**
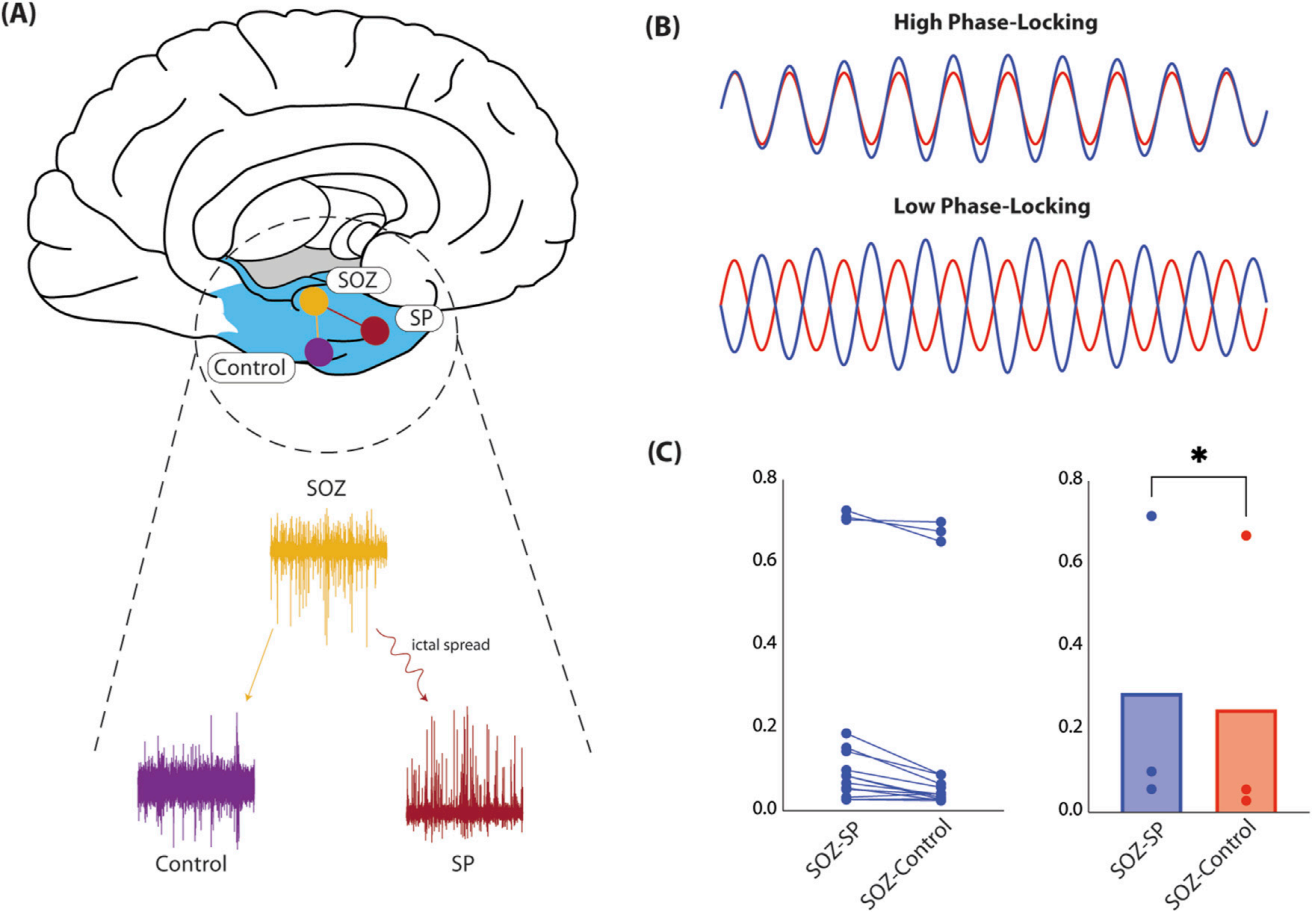
Functional connectivity in human epilepsy patients. **(A)** Here, we show the schematic for how FC was computed in our cohort of DRE patients. Specifically, we considerd icEEG obtained during neuromonitoring for seizure localization. We selected for contacts recording from regions identified as SOZ, SP, or control, equidistant to the SOZ. We then computed PLC and compared across all SOZ-SP and SOZ-control contact pairs across all patients (*left*). **(B)** Here, we depict extreme cases of what PLC measures. Specifically, PLC captures how in-phase, or synchronized two signals are. High PLC values (*top*) mean signals are synchronized, and therefore suggests that the underlying recording sites may be functionally connected. On the contrary, low PLC values recording sites are likely unrelated to one another. **(C)** We average PLC computed for all SOZ-SP and SOZ-control contact pairs. We show that within a given contact pair, SOZ-SP contacts are more functionally connected than their respective SOZ-control pair (*left*). When we compare PLC across all patients, we find that SOZ-SP contact pairs are truly significantly more synchronized, and hence likely more strongly connected, compared to SOZ-control pairs (0.29 *versus* 0.25, *t*(2) = 6.72, *p* = 0.0215, paired t-test, *right*). Abbreviations: FC, functional connectivity; DRE, drug-resistant epilepsy; SOZ, seizure-onset zone; SP, seizure spread; icEEG, intracranial electrocorticography; PLC, phase-locking coherence*.*

For each interictal epoch, we considered contacts recording from regions of seizure onset (SOZ), spread (SP), and uninvolved controls (Figure 1A). We were interested in comparing FC between SOZ-SP and SOZ-control. For each contact, we preprocessed signals by applying a local detrending procedure to remove slow fluctuations from the time series. We then used a regression-based approach to remove line noise at 60 and 120 Hz (Kutsy, 1999). We used a low-pass type 1 finite impulse response (FIR) filter (order = 180), to remove higher order line harmonics as well as high-frequency noise. After preprocessing signals, we applied a band-pass FIR filter (order = 390) designed using the Parks-McClellan algorithm in the high-gamma band (70–90 Hz) (Parks and McClellan, 1972). We were interested in the high-gamma band considering its relevance in seizure onset regions (Ren et al., 2015). We computed phase-locking coherence (PLC) based on raw real Hilbert transformed time series, comparing estimates across SOZ-SP and SOZ-control pairs (Lachaux et al., 1999). We considered PLC as our FC metric as it measures the degree of synchronization between icEEG signals, with high values reflecting greater synchronization, and this connectivity, and low values reflecting weaker (Figure 1B).

### Neuron-glia culture preparation

We established a mixed neuron-glia rat cortical cell culture on MEAs according to previously published protocols (Ksendzovsky et al., 2022; Mortazavi et al., 2022). Cortices were dissected from newborn P1 rat pups in a modified Puck’s dissociation medium D1 (5 mM HEPES, 16.5 mM glucose, 22 mM Sucrose, 137 mM NaCl, 0.32 mM ${Na}_{2}$ HPO_4_, 0.22 mM KH_2_PO_4_ in deionized water, pH 7.4, Osm 320-330). Cultures typically consisted of cortical cells harvested from 3 to 12 pups (male or female). Once cortices were collected, cortical cells were subsequently dissociated in a Puck’s/papain solution (1.5 mM CaCl_2_, 0.5 mM EDTA, 0.75 % papain (Worthington Biochemical Corporation, Lakewood, NJ) and 8.25 nM Cysteine in D1 medium). After appropriate dissociation, cortical cells were plated on MEA plates precoated with 1 mg/mL of poly-D-lysine (PDL) in borate buffer, pH 8.4. Cells were plated in 6-well Axion CytoView MEA plates (Axion Biosystems, Atlanta, GA) at a density of $2\times 10^{5}$ cells/well, with each well serving as a technical replicate. Cultured plates were maintained in a cell-culture incubator at 37° C and 5 % CO_2_. 24-h after plating, a complete media change was performed, after which cells were maintained in maintenance medium (5 % FBS, 1X B-27, 1X antibiotic-antimycotic mix, 5 mM HEPES, 1.2 mM L-glutamine in Neurobasal medium, pH 7.4) with partial media changes every 48-h. To reduce neuron-glia culture variability, rat pup cortices are mixed after dissection and prior to plating on MEA wells. Furthermore, neuron-glia cultures are obtained from multiple rat moms, to further reduce inter-MEA well variability.

### Induction of hyperexcitable activity using 4-AP

There are numerous techniques to capture hyperexcitable activity in dissociated cultures on MEAs (Grainger et al., 2018). One approach is introduction of proconvulsant agents (ex. Picrotoxin, Gabazine, PTZ, Bicuculline, Tutin, Tranexamic Acid, Endosulfan, 4-Aminopyridine (4-AP), SNC80, NMDA, Linopirdine, Strychnine HCL, Amoxapine, Pilocarpine HCL, Thioridazine HCL, Domoic Acid, Tetrodotoxin (TTX)) (Bradley et al., 2018). Another is by manipulation of ion concentrations (ex. low ${Mg}_{2}^{2+}$ ) (Bradley et al., 2018). In this model, we used 4-AP, a potassium-channel blocker that has been well established as a reliable and potent seizurogenic agent to model seizures *in vitro* (Bradley et al., 2018; Choquet and Korn, 1992; Cramer et al., 1994; Yamaguchi and Rogawski, 1992; Pea and Tapia, 2000; Gonzalez-Sulser et al., 2011). Specifically, 4-AP results in partial blockade of repolarizing A-type potassium channels, resulting in membrane depolarization, increased intracellular ${Ca}^{2+}$ concentrations, and increased glutamate release from presynaptic terminals, ultimately inducing hyperexcitable activity (Žiburkus et al., 2013; Yokoi et al., 2022). We chose 4-AP over other candidate models, such as low ${Mg}_{2}^{2+}$ or electrical stimulation, for its relative simplicity of use and minimization of confounding effects. Namely, 4-AP can be delivered by adding a low volume of concentrated stock solution to treatment MEA wells to achieve the desired working concentration. Furthermore, 4-AP has been shown to induce slow seizure-like events (SLEs), unlike low ${Mg}_{2}^{2+}$ and electrical stimulation models (Heuzeroth et al., 2019; Luhmann et al., 2000; Chiang et al., 2018).

**FIGURE 2:**
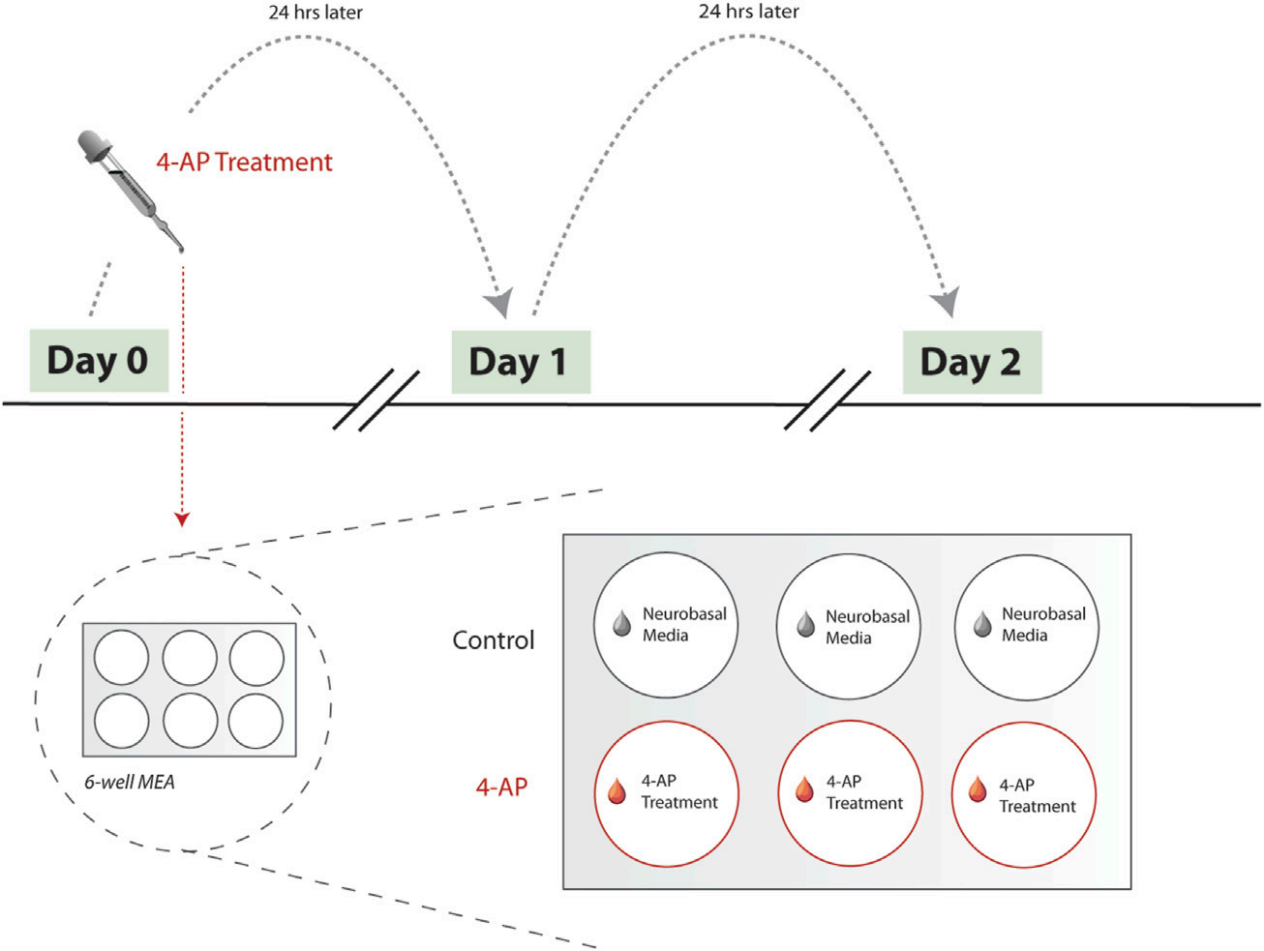
Experimental workflow. Here, we show the experimental workflow, delineating 4-AP treatment protocol. Treatment begins Day 0, after MFR has stabilized across neuron-glia cultures (see *Methods*). MEA recordings are collected prior to each day’s 4-AP wash-in using the Maestro Pro MEA system (Axion Biosystems, Atlanta, GA). 6-well MEA plates are used, with three wells serving as untreated controls and remaining used for 4-AP treatment. Protocol was repeated across multiple biological replicates, with each 6-well MEA plate reflecting a single biological replicate. During a single day’s 4-AP treatment, treatment wells (*red*) were spiked with concentrated stock solution of 4-AP. Simultaneously, control wells (*grey*) were treated with equivalent volume of Neurobasal medium. Treatment period lasts for 30-min, after which all wells (control and 4-AP-treated) undergo full wash-out and media change with Neurobasal medium. Treatment is repeated for 2 days, at approximately the same time each day. As such, each MEA recording is effectively collected 24-h apart, allowing for quantifying chronic changes. Abbreviations: 4-AP, 4-aminopyridine; MFR, mean firing rate; MEA, microelectrode arrays.

Experiments with 4-AP began after neuron-glia culture maturation and differentiation, around day-*in-vitro* (DIV) 20, as suggested by previous literature (Latchoumane et al., 2018) and direct observation of stabilized mean firing rate (MFR) across at least 3 days. Experimental protocol consisted of obtaining 5-min baseline MEA recordings prior to 4-AP treatment. After baseline MEA recordings, concentrated 4-AP solution was applied to treatment wells, achieving a 500 μ M working concentration of 4-AP. This was chosen as the most optimal 4-AP concentration to achieve hyperexcitable activity in our *in vitro* model after dose response experiments and considering it is concordant with ranges typically used *in vitro* (Matsuda et al., 1986). Simultaneously, control wells were “treated” with an equivalent volume of Neurobasal medium. 4-AP treatment wells were exposed to 4-AP-induced hyperexcitable conditions for 30-min. Afterwards, treatment and control wells underwent full media changes. This protocol was repeated for a total of 3 days, to allow for study of changes on a chronic timescale. Effectively, other than the initial MEA recording, subsequent MEA recordings are collected 24-h after previous day’s 4-AP treatment (Figure 2).

### MEA recording acquisition and preprocessing

*In vitro* electrophysiology was collected using the Maestro Pro MEA system (Axion Biosystems, Atlanta, GA) and AxIS Navigator software v3.5.2 (Axion BioSystems, Atlanta, GA). We used six-well MEA plates, due to their high microelectrode density (64 microelectrodes). Microelectrodes are made of polymer poly (3,4-ethylenedioxythiophene) (PEDOT) and arranged in an 8 × 8 grid, with a 50 μ m electrode diameter and 300 μ m interelectrode spacing. All voltage traces were sampled at 12.5 kHz.

To extract spiking activity, we processed all electrophysiology recordings offline using custom MATLAB (MathWorks, Natick, MA) scripts. Preprocessing steps involved downsampling signals from 12.5 kHz to 2.5 kHz and band-pass filtering signals between 0.1 and 300 Hz (Butterworth, order = 20). These steps were done to eliminate the impacts of low-frequency drift, DC artifacts, and filter for multi-unit activity (MUA) to capture high-frequency neuronal spiking (Stark and Abeles, 2007; Waldert et al., 2013; Ahmadi et al., 2021). Subsequently, signals were filtered using a series of notch filters (Butterworth, order = 4) to eliminate 60 Hz line noise and its first two harmonics (i.e., 120, 180 Hz), with a narrow bandwidth within 1 Hz of the target frequency. All signals were subsequently *z*-scored.

### Functional connectivity

We were interested in computing changes that estimate synchronized neural activity, or FC, across all multi-unit populations. To this end, we considered degree of correlation, a measure of statistical interdependence, between spike trains (Cohen et al., 2002; Selinger et al., 2004; Chiappalone et al., 2006; Chiappalone et al., 2011; Eisenman et al., 2015). For each spike train recorded from respective microelectrodes, we vectorized spike counts by binning spikes in non-overlapping 50-ms windows. FC was then quantified by computing Pearson’s correlations (Fisher *z*-transformed) across all unique pairwise binned spike trains in each well.

Of note, considering our choice of Pearson’s correlation (Fisher *z*-transformed) across pairs of binned spike trains, FC estimates would be biased when including periods of highly-synchronous firing, such as that observed during population bursts. This is a well understood confounder, in which correlation as a measure of spike train synchrony is positively biased in periods of higher firing rate (Cutts and Eglen, 2014). To assess whether inclusion of population burst periods in FC analysis would truly bias estimates in our data, we compared impact of including and excluding population burst periods on FC estimates. We observed in representative MEA recordings that FC was significantly greater when including burst periods, as anticipated ( ρ = 0.038 *versus* 0.018, *p* < 0.0001, two-sample *t*-test). This suggests that including highly synchronous burst periods would bias our FC estimates, and hence, our decision to exclude population burst periods.

### Mapping network connectivity in low-dimension space

The aim of the presented method is to model seizure activity and be able to characterize network changes. To this end, we compute FC, as described above, to estimate connectivity between pairwise multiunit populations. However, network dynamics can be complex and therefore may not be appropriately captured by simply comparing difference of means of computed FC estimates. For example, if a treatment group exhibits a small number of pairwise connections that are preferentially strengthened, this difference may not be captured by comparing difference of means between treatment and control groups as connectivity distributions may be skewed. As such, it is important we devise a method that is able to capture relevant network changes.

In recent years, dimensionality reduction has emerged as a powerful tool for revealing patterns in complex neural data (Langdon et al., 2023; Cunningham and Yu, 2014). Here, we use the high-dimensional connectivity information we compute and represent this in a low-dimension embedding, using principal components analysis (PCA), allowing us to characterize how network connectivity evolves across a single well, over time.

We performed dimensionality reduction separately for each well considering each individual well has unique network connections that may evolve differently over time. Conducting dimensionality reduction across different wells represented in the same feature matrix would confuse the dimensionality reduction procedure into treating each pairwise connectivity estimate as a feature representing the same relative information across different wells, when in reality, pairwise connectivity estimates only serve as meaningful features in the context of individual wells.

We constructed a single *m* × *k* feature matrix (***A***) for each well, where *m* = number of MEA recordings (i.e., 3), *k* = $\left(\begin{array}{c} n\\ 2 \end{array}\right)$ , representing the number of unique microelectrode pairs, and *n* = number of microelectrodes (i.e., 64). This results in the following feature matrix:

$$A=\left[\begin{array}{c} \alpha_{1,\left(i,j\right)}\;\cdots \;\alpha_{1,\left(n,n-1\right)}\\ \vdots \;\ddots \;\vdots \\ \alpha_{m,\left(i,j\right)}\;\cdots \;\alpha_{m,\left(n,n-1\right)} \end{array}\right]$$

where (*i, j*) $\in \;Z^{+}\;|\;1\leq \left(i,j\right)\leq n$ and $\alpha_{\left(i,j\right)}$ = FC estimate between microelectrode *i* and *j*. After performing PCA analysis, we extract the low-dimension embedding for each recording timepoint using the first two principal components (PCs). We plot PC2 *versus* PC1, to visually represent how network connectivity evolves in a well over time. This results in three points plotted in a low-dimension space, with each point corresponding to the network connectivity in a well, across pre-treatment, Day 1, and Day 2 recordings. To assess how significantly network connectivity changes, we compute Euclidean distance of points to the pre-treatment point. Points that remain in a smaller subspace likely reflect similar network connectivity patterns, whereas those that occupy distinct subspaces reflect vastly different network connectivity patterns.

### Statistical analyses

All data analysis was conducted using custom MATLAB scripts (MathWorks, Natick, MA) and Prism (GraphPad, San Diego, CA). Groups were compared using two-tailed *t*-tests. Parametric statistical tests were used considering data were normally distributed (*p* > 0.05, D’Agostino-Pearson test). We corrected for multiple comparisons using *post hoc* Holm-Bonferroni testing, where appropriate (Curran-Everett, 2000). A threshold of *p* < 0.05 was used to denote statistical significance. Asterisks (*), (**), (***), and (****) indicates significance *p* < 0.05, *p* < 0.01, *p* < 0.001, *p* < 0.0001, respectively. Data are represented as mean $\left(\bar{x}\right)$ ± standard error of mean (SEM), unless otherwise noted.

## Results

### Evidence of seizure networks in epilepsy patients

To investigate seizure networks in epilepsy, we computed FC between regions involved in seizure onset and primary spread, and compared this to FC between seizure onset and equidistant, uninvolved control regions. We hypothesized that areas connected by seizure spread are more strongly connected compared to those that are not, lending evidence to the network theory of epilepsy. In our patient cohort, we find that regions involved in seizure spread are in fact more strongly synchronized in the high-gamma band (70–90 Hz), compared to control regions (0.29 *versus* 0.25, *t* (2) = 6.72, *p* = 0.0215, paired *t*-test, Figure 1C). From this, it is clear that on average, SOZ and SP regions are more synchronized than SOZ and control regions, suggesting that regions of seizure activity are more strongly functionally connected, and may comprise a pathologic seizure network that strengthens over time, serving as the primary motivation for our *in vitro* seizure model.

### 4-AP captures hyperexcitability *in vitro*

**FIGURE 3:**
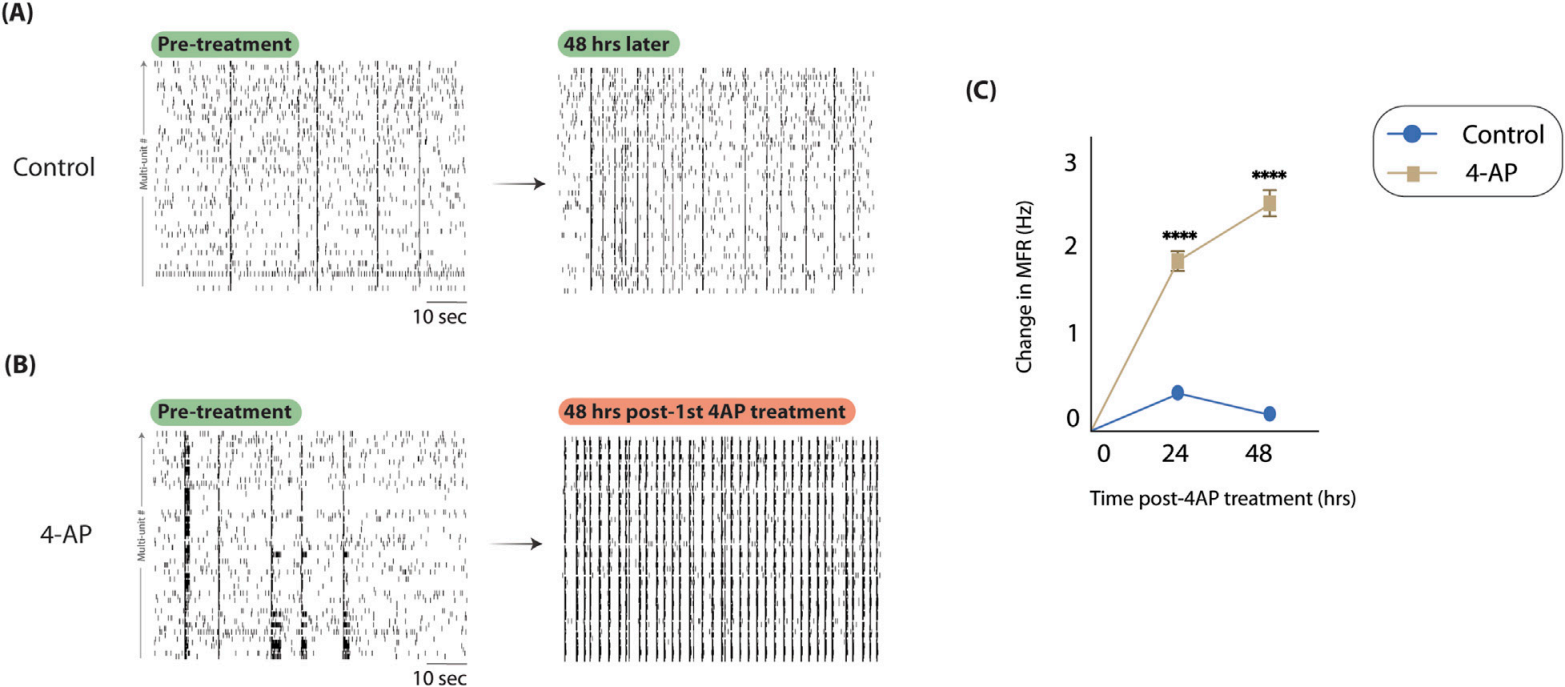
4-aminopyridine successfully captures hyperexcitable activity *in vitro*. To assess if 4-AP could capture hyperexcitability in our neuron-glia cultures on MEAs, we considered spike rasters and MFR before and after chronic 4-AP treatment. **(A)** Here, we show spike rasters from a representative control well before 4-AP treatment protocol (*left*) and after (*right*). We observe no discernable changes in spiking density. **(B)** Here, we consider spike rasters before (*left*) and after (*right*) 4-AP treatment. Compared to control, we note significantly higher spiking density and more synchronous firing periods, consistent with expectations. **(C)** We show changes in MFR throughout 4-AP treatment protocol, normalizing to pre-treatment MFRs, across multiple technical (9) and biological (3) replicates. MFR is significantly moreso increased in 4-AP-treated wells compared to control after just 1 day of treatment (1.90 ± 0.10 *versus* 0.53 ± 0.04, *t* = 11.70, Cohen’s *d* = 0.73, *p* < 0.0001, two-sample *t*-test). MFR elevation persisted after 2 days of 4-AP treatment as well (2.51 ± 0.14 *versus* 0.31 ± 0.05, *t* = 14.36, Cohen’s *d* = 0.89, *p* < 0.0001, two-sample *t*-test). Data are represented as $\bar{x}\;\pm$ SEM. Abbreviations: 4-AP, 4-aminopyridine; MFR, mean firing rate.

**FIGURE 4:**
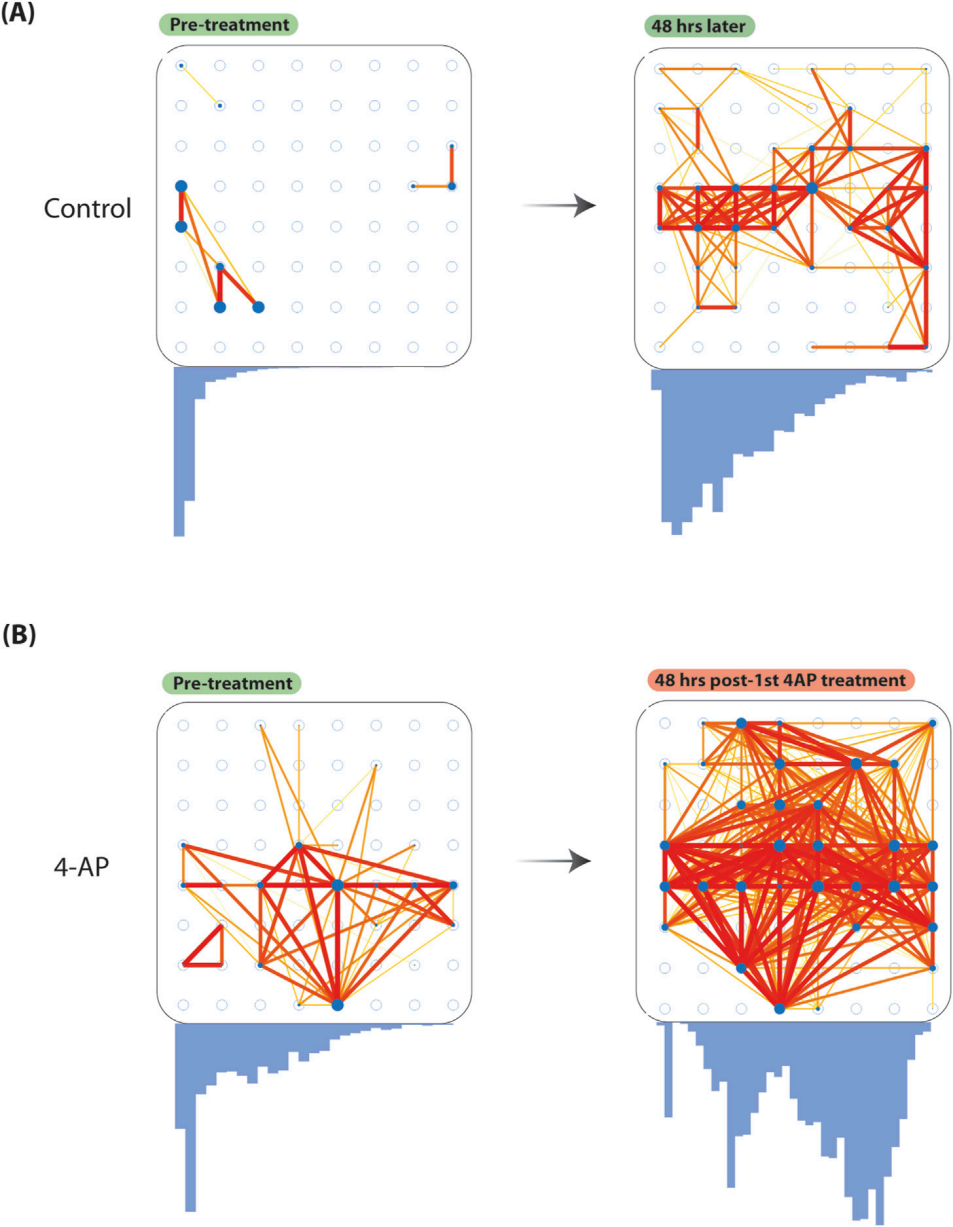
Increased baseline functional connectivity after chronic 4-aminopyridine treatment. To show our model can be used to study network changes, we considered FC changes before and after chronic 4-AP treatment. We quantified FC considering pairwise Pearson’s correlation (Fisher *z*-transformed ρ ) across binned spike trains across all unique microelectrode pairs (see *Methods*). We create network plots by plotting significant pairwise connections (Fisher *z*-transformed ρ> 0.80). Stronger connections (i.e., edges) are delineated using warmer colors (*red*) lines that are thicker. Weaker connections (i.e., edges) are delineated using cooler colors (*yellow*) lines that are thinner. Microelectrodes (i.e., nodes) with more connections are delineated by *blue* with larger diameter. Below each network plot, histogram distributions of Pearson’s correlations (Fisher *z*-transformed ρ ) are shown. **(A)** Here, we show changes in FC in a representative control well from before (*left*) experimental protocol and after (*right*). We observe that FC estimates increase slightly after 48-h, and maintain a unimodal distribution (*blue histograms*). **(B)** Here, we show changes in FC in a representative 4-AP well before (*left*) and after (*right*) chronic 4-AP treatment. We observe a discernable increase in the number of stronger pairwise connections, compared to control. Furthermore, FC increases significantly more in 4-AP-treated wells, compared to control ( Δρ CI(95 % ) = [0.3827, 0.4155], *t* = 47.75, *p* < 0.0001, two-sample *t*-test). Interestingly, FC correlation distributions become more bimodal after chronic 4-AP treatment as well. Abbreviations: FC, functional connectivity; 4-AP, 4-aminopyridine.

The presented model is predicated on effectively capturing hyperexcitable conditions *in vitro* to model high firing conditions observed during seizures. To assess whether 4-AP-induced hyperexcitability was reflective of this, we considered spike rasters and changes in neuronal firing activity. When considering representative spike rasters from control (Figure 3A) and 4-AP-treated (Figure 3B) and wells, apparent differences in firing activity are observed, with the 4-AP-treated well exhibiting increased spiking density and periods of synchronous firing (bursts). Changes in firing activity were quantified using mean firing rate (MFR) normalized to pre-treatment baseline (i.e., Day 0, see Figure 2). We observed that after just one treatment with 4-AP, the next day’s MFR was significantly elevated (1.90 ± 0.10 *versus* 0.53 ± 0.04, *t* = 11.70, Cohen’s *d* = 0.73, *p* < 0.0001, two-sample *t*-test, Figure 3C). In fact, MFR was consistently elevated after 2 days of 4-AP treatment at baseline as well (2.51 ± 0.14 *versus* 0.31 ± 0.05, *t* = 14.36, Cohen’s *d* = 0.89, *p* < 0.0001, two-sample *t*-test, Figure 3C). In conjunction, these findings validate that 4-AP is effective in capturing hyperexcitable conditions that persist at baseline, creating a chronic model of epilepsy, ultimately allowing for study of longitudinal network changes.

### Increased functional connectivity after chronic 4-AP treatment

To show the presented model effectively captures network changes, we compared functional connectivity (FC) (Pearson’s correlation, Fisher *z*-transformed ρ ) changes before and after chronic 4-AP treatment. We observe that prior to 4-AP treatment (Day 0), FC estimates are similarily distributed between 4-AP and control wells and not significantly different (Figures 4A, B). After 2-days of 4-AP treatment, we note FC correlations are significantly increased in 4-AP-treated wells, compared to control ( Δρ CI(95 % ) = [0.3827, 0.4155], *t* = 47.75, *p* < 0.0001, two-sample *t*-test). Furthermore, we observe that while FC correlation distributions remain unimodal in control wells after 2-days, 4-AP-treated wells show FC correlations forming a bimodal distribution. We additionally plotted the strongest pairwise connections (Fisher *z*-transformed ρ> 0.80) across 4-AP and control wells, and show that there are far greater strong connections after 2-days of 4-AP treatment, compared to control.

### Network connectivity occupies distinct subspace after 4-AP treatment

**FIGURE 5:**
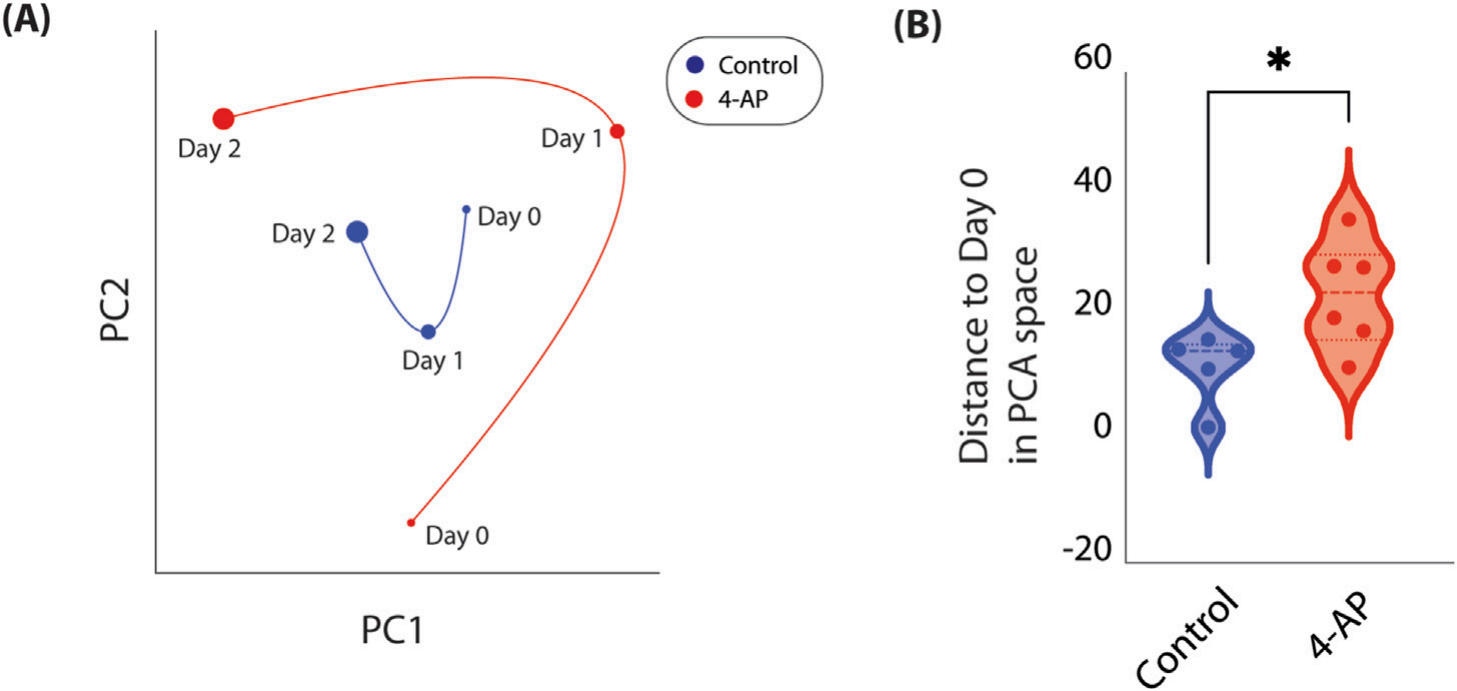
Temporal evolution of network connectivity mapped in low-dimension space. As network connectivity may be complex and change dynamically, we developed a method to track how a well’s network connectivity evolves over time by reducing high-dimensional connectivity information to a low-dimension embedding (see *Methods*). **(A)** Here, we show how network connectivity evolves across 3 days in representative control (*blue*) and 4-AP-treated (*red*) wells. Timepoint represented is delineated next to each point. We observe that after 2 days of chronic 4-AP treatment, baseline network connectivity occupies a distinct subspace, far from pre-treatment network connectivity. In contrast, we observe that network connectivity in a representative control well occupies a smaller subspace, closer to what is observed Day 0. **(B)** To quantify change in network connectivity over time, we compute the Euclidean distance of each point to Day 0. We find that after 2 days, compared to controls, 4-AP-treated wells show significant changes in network connectivity compared to pre-treatment (22.43 ± 3.55 *versus* 10.67 ± 5.74, *t* = 2.58, Cohen’s *d* = 1.43, *p* = 0.0298, two-sample *t*-test). Abbreviations: FC, functional connectivity; 4-AP, 4-aminopyridine.

We reduced high-dimensional functional connectivity information to a low-dimension embedding to allow for study of how a individual well’s network connectivity evolves over time (see *Methods*). Using this method, we are able to plot how a well’s network connectivity is evolving over time and compare network connectivity changes across different wells. We do so across all control and 4-AP-treated wells. Specifically, we observe that network connectivity patterns in control wells tend to cluster around pre-treatment observations, suggesting that networks are relatively stable, and unchanged over 2 days of recordings (Figure 5A). However, when we consider 4-AP-treated wells, we observe that after one and 2-days of 4-AP treatment, network connectivity patterns deviate from what is observed pre-treatment, suggesting that 4-AP causes network changes over time (Figure 5A). We quantify these network changes by computing distance of points to Day 0 recordings, on the low-dimension space. We find that after 2 days of chronic 4-AP treatment, network connectivity patterns occupy a unique subspace distinct from pre-treatment, compared to control (22.43 ± 3.55 *versus* 10.67 ± 5.74, *t* = 2.58, Cohen’s *d* = 1.43, *p* = 0.0298, two-sample *t*-test, Figure 5B).

## Discussion

A significant challenge in studying neurologic disease is the availability of representative models. Although it is most optimal to study disease pathology at the patient level, oftentimes it is not possible to truly understand the pathomechanisms of a disease without a fundamental understanding of underlying mechanistic changes. *In vivo* and *in vitro* models offer the ability to study principal changes in the context of representative models of disease, greatly contributing to our understanding of them.

Epilepsy is a common neurological disorder, however, very little is understood of the mechanisms surrounding epileptogenesis. Until recently, epilepsy has largely been seen as a focal disorder, with seizure models reflecting this. However, over recent years, we have come to understand epilepsy as a network disorder, recruiting various cortical and subcortical regions into a pathologic seizure network (Kramer and Cash, 2012; Scharfman et al., 2018; Piper et al., 2022; Bröhl et al., 2023). Numerous intracranial studies considering network connectivity patterns have lent support to this idea, some even suggesting that disruption of seizure networks is likely critical in achieving seizure-freedom (Andrews et al., 2019; Neal et al., 2020; Jehi, 2017; Rijal et al., 2023; Taylor et al., 2018; Josephson et al., 2013).

In fact, here, we show evidence of high interictal FC between areas linked by seizure spread. These findings lend direct support for the network theory of epilepsy, suggesting that seizure activity travels along more strongly connected neuronal populations. This corroborates previous findings in support of the network theory of epilepsy and further suggests that longitudinal network changes are likely involved in seizure network formation, resulting in baseline changes that can be observed interictally. However, icEEG studies considering seizure networks only provide snapshots in time, and preclude the ability to study longitudinal network development. As such, it is imperative we have models that allow us to investigate network changes over time in the context of epileptogenesis. However, to date, there are no seizure models that consider network changes, with most simply focusing on studying molecular changes underlying hyperexcitability or focal alterations. As such, in this study, we created a novel *in vitro* seizure model in order to model changes in neuronal network connectivity associated with epileptogenesis, motivated by our human epilepsy findings.

As previously described, here we show 4-AP chronically increases neuronal firing, akin to an *in vitro* correlate of kindling, where residual hyperexcitability may lead to compensatory and pathologic changes within epileptic networks (Heuzeroth et al., 2019; Leite et al., 2005; Lillis et al., 2015). In addition, we demonstrate that 4-AP induces FC changes, likely secondary to induced hyperexcitability, which may replicate the changes that occur in human epileptic networks, providing a bridge between experimental models and clinical epilepsy. We further show changes in network activity using a dimensionality reduction-based method to characterize network connectivity changes in MEA wells.

Because network dynamics are complex, FC may fail to appropriately capture changes that could be informative of strengthening or weakening of specific pairwise connections. Additionally, it is challenging to capture complex network changes using a single measurement. As such, we reduced high-dimensional pairwise FC information into a low-dimension embedding, from which network connectivity information for an individual well can be simply visualized and changes across time can be quantified. Using this, we show changes in wells’ network connectivity over time and find that these results parallel changes observed when considering FC alone, suggesting that this method retains information conveyed by traditional FC measures. Ultimately, we believe using dimensionality reduction to reduce network connectivity information allows for more accurate capture of how a MEA well’s connectivity patterns change over time.

While exisiting *in vitro* models capture aspects of epileptic activity, to the best of our knowledge, none allow for the detailed study of chronic network changes of seizure-like states as effectively as our proposed method. Our protocol holds many advantages, including the unique ability to study network changes and circuit reorganization afforded by the composition of dissociated neuron-glia cultures. The inclusion of both neurons and their supporting glial cells not only better represents the *in vivo* environment, but also provides a more adaptable experimental setup with enhanced survival and cellular development (Kaech and Banker, 2007). This flexibility is crucial for investigating network dynamics in the absence of preexisting connections, allowing for precise manipulations and observations of how epileptic networks evolve and respond to interventions. For example, recent studies have suggested that specific regulatory pathways are altered in epileptic tissue (Ksendzovsky et al., 2022; Hammer et al., 2019; Pfisterer et al., 2020; Sumadewi et al., 2023; Meng et al., 2015). Using our model, we can define the molecular basis of network changes by introducing small molecular inhibitors that target these pathways and investigate subsequent network changes.

Nevertheless, this model’s reliance on initially naïve networks may also be seen as a limitation, given that seizures in humans affect preexisting neural networks. To address this, we begin our 4-AP treatments post-culture maturation, aiming to more accurately represent changes observed in human cortical/subcortical networks. An additional limitation of our *in vitro* model is the inherent inability to accurately replicate epileptiform activity, as is possible in more physiologically accurate animal models of epilepsy (i.e., electrical kindling, Kainic Acid). Our model uses 4-AP, a potassium-channel blocker that impairs neuronal repolarization and hyperpolarization, this increases overall neuronal excitability in the network allowing for modeling of seizure-like conditions. However, it is important to note that potassium-channel blockade does not underly representative seizure activity seen in chronic models of epilepsy i.e., Kainic Acid animal models of epilepsy, which demonstrate spontaneous recurrent seizures. Thus, it is important to restate that our model captures hyperexcitable neuronal firing activity which is observed as a result of seizure-like activity, and not direct seizure activity (Wong, 2011). As such, *in vitro* models preclude the ability to characterize true ictal activity, and rather limit us to correlates such as neuronal firing activity (i.e., firing rate, bursting rate). These are well-acknowledged limitations of *in vitro* epilepsy models, as such, conclusions must be considered in this context (Oblasov et al., 2024). However, despite this, our model still allows for study of what may happen to neuronal networks under hyperexcitable conditions, such as those captured by seizure activity. A key hallmark of epilepsy is repeated seizure insults which induce hyperexcitable conditions in cortical/subcortical regions. Our 4-AP model was created to simulate chronic changes that occur in the context of repeated seizure events, reflective of what happens in epilepsy *in vivo*. Daily 4-AP treatments not only replicate kindling activity, but also provide a mechanism by which to simulate the temporal progression of changes associated with epileptogenic stimuli. While many theories of epileptogenesis exist, hyperexcitable onslaughts leading to maladaptive network changes in a kindled fashion is certainly among them (Bromfield et al., 2006; Marques et al., 2022). As such, it is certainly plausible to use our 4-AP model to understand how changes in network activity advance throughout epileptogenesis. In the future, this *in vitro* model and its network findings can be corroborated by *in vivo* animal studies using high-density MEAs implanted directly on the cortical surface. Recent groups have shown that Utah Electrode Arrays (UEAs) (Blackrock Microsystems, Salt Lake City, UT) can be implanted directly on the cortical surface of adult rats, allowing for both multi-unit and single-unit recordings (Black et al., 2018; Nolta et al., 2015). Using this, similar to our *in vitro* model, high-resolution neuronal network connectivity patterns and changes can be characterized in the context of a more representative animal epilepsy model.

Additionally, our method’s integration of MEA recordings enable investigation of multi-unit populations, offering insights that are unattainable with traditional patch-clamp techniques used in slice models, which do not capture population-level dynamics. The use of microelectrodes in our model allows for capture of extracellular field potential at the level of multi-unit populations, enhancing our ability to characterize network dynamics, synaptic transmission, and plasticity in the *in vitro* setting (Hofmann and Bading, 2006).

Studying mechanistic network-level changes is crucial to advancing our pathophysiologic understanding of epilepsy and guiding therapeutic interventions. Here, we show that there likely exist interictal functional seizure networks in a cohort of DRE patients. We then show how these seizure networks may be modeled using *in vitro* neuron-glia cultures plated on MEAs. Given recent studies suggesting that disruption of seizure networks is important in achieving seizure control, our findings and *in vitro* model open a range of possible future interventions. For example, considering the persistence of seizure networks interictally, icEEG may be used to characterize critical nodes in a seizure network to guide subsequent surgical intervention (ex. resection, neuromodulation). An additional therapeutic application from our model is the optimization of neuromodulation for targeting seizure networks. Neuromodulation is an effective treatment option that uses brief pulse stimuli (BPS) to reduce seizure activity prior to its full-blown onset. Although offering a new and improved therapeutic approach to seizure control, neuromodulation still fails to achieve seizure control in a subset of patients (Boddeti et al., 2022). Recent studies suggest that this treatment failure may be due to underlying network connectivity patterns, which may inform those who may benefit from neuromodulation and those who may not. To this end, characterizing a patient’s interictal seizure network connectivity patterns using icEEG may inform those who may benefit from neuromodulation *versus* those who may not. Additionally, recent studies suggest that neuromodulatory devices exerts their therapeutic effects by modulating network activity, as opposed to focal activity. As such, our presented *in vitro* MEA model offers a unique platform in which this hypothesis can be tested, as MEA technology allows for applying direct electrical stimulation. Furthermore, this allows for testing various neuromodulation paradigms and their effects in 1) modulating network activity and 2) controlling seizure-like activity. Ultimately, using MEA technology to better understand network activity in seizure models allows for optimization of preexisting treatments such as neuromodulation and also discovery of new ones, by allowing for study of specific pathway inhibitors and pharmacologic interventions on network connectivity changes.

## Conclusion

Here, we present a novel *in vitro* model to study seizure networks using neuron-glia populations cultured on MEAs. We show that our 4-AP model serves as a robust *in vitro* tool for modeling connectivity changes associated with epileptogenesis. This model captures not only acute hyperexcitablity, a hallmark of epileptic networks, but also chronic network adaptations over time, as evidenced by increased FC of underlying neuronal networks. Furthermore, we introduce a novel method for characterizing aggregate network connectivity and how it changes over time. We believe that our model and methods effectively capture network dynamics akin to those in epilepsy patients. Considering the recent idealogical shift and strong evidence for epilepsy as a network disorder, ultimately, we believe that our model can be used to not only better understand epileptogenesis, but also begin to develop new therapeutics, and adapt preexisting ones (i.e., neuromodulation) to target pathologic seizure networks.

## Data Availability

The raw data supporting the conclusions of this article will be made available by the authors, without undue reservation.
